# Unravelling the Interaction Mechanism Between Oryzanol and Human Serum Albumin: An Integrated Approach Using Multispectral Analysis and Molecular Simulations

**DOI:** 10.3390/foods15081420

**Published:** 2026-04-18

**Authors:** Chenge Zhang, Siyi Zhu, Shanshan Duan, Keyu Meng, Menglin Guo, Lanlan Wei, Jiayi Shi, Fuqiang Liang

**Affiliations:** 1 College of Food Science and Engineering, Collaborative Innovation Center for Modern Grain Circulation and Safety, Nanjing University of Finance and Economics, Nanjing 210023, China; zcgzcgzcg2023@163.com (C.Z.); 15850567521@163.com (S.Z.); dss9307@163.com (S.D.); mengkeyu2022@163.com (K.M.); guomenglin0314@163.com (M.G.); 2 College of Food Engineering, Anhui Science and Technology University, Chuzhou 233100, China; 18356130656@163.com

**Keywords:** oryzanol, human serum albumin, non-covalent interaction, multispectral, molecular simulation

## Abstract

Gamma-oryzanol (Ory), a major bioactive constituent of rice bran oil, has attracted increasing attention because of its antioxidant and cholesterol-lowering properties. In this study, the interactions between Ory and human serum albumin (HSA) and the underlying molecular mechanisms were investigated. The quenching of HSA fluorescence by Ory occurred via a mixed mechanism, indicating the formation of a stable complex. Thermodynamic analyses and molecular dynamics showed that the HSA-Ory complex was stabilised primarily by hydrogen bonding and van der Waals interactions. Moreover, competitive site marker experiments, complemented by molecular docking and MM-PBSA calculations revealed that Ory specifically targets site I of HSA, engaging in stable interactions with critical residues such as Trp214 and Lys199. Additionally, the dissociation behaviour of Ory was explored using steered molecular dynamics simulations, highlighting the role of specific amino acid residues in regulating the dissociation of Ory from HSA site I. Overall, this study provided molecular insights into the binding mechanisms and interactions between HSA and Ory.

## 1. Introduction

Human serum albumin (HSA) is a highly abundant globular plasma protein composed of 585 amino acid residues that plays a pivotal role in transporting a wide range of endogenous and exogenous compounds [1]. It binds reversibly to numerous bioactive molecules primarily through non-covalent interactions [2,3]. Structurally, HSA consists of three homologous domains (I–III), each of which is subdivided into two subdomains (A and B). The two principal ligand-binding sites, commonly designated as site I and site II, are located in subdomains IIA and IIIA, respectively [2]. These sites enable HSA to bind and transport various bioactive small molecules with diverse structures, such as phenolic acids [4], stilbenes [5], flavonoids [6] and alkaloids. Studies have indicated that the binding of bioactive compounds to HSA can induce conformational alterations that may affect its binding capacity to other ligands [7]. Because this binding is reversible, structural changes in HSA may also regulate the release of bioactive substances. Moreover, the distribution and bioavailability of bioactive substances could be markedly influenced by their binding to HSA. HSA typically improves the solubility of bioactive substances in the plasma, enhances their stability, and regulates their delivery [8]. Therefore, understanding the interactions between HSA and its ligands is essential.

**Figure 1:**
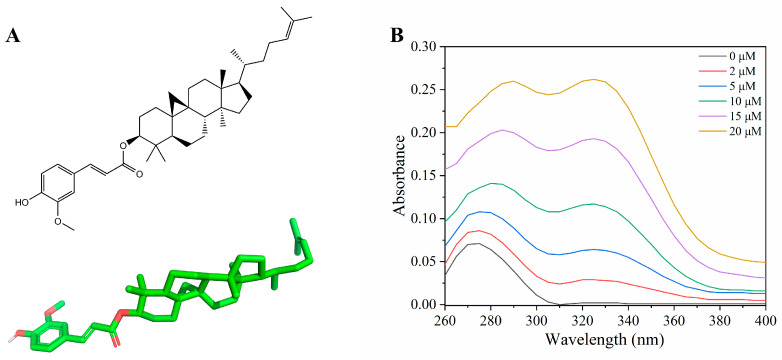
The 2D and 3D structures of Ory (**A**). UV-Vis spectra of HSA-Ory complex (**B**).

Gamma-oryzanol (Ory) (Figure 1A) is an important bioactive compound that is abundant in cereals such as rice [9]. Numerous studies have demonstrated that it shows a wide spectrum of health-promoting effects including antidiabetic, antioxidant, anti-ageing, and anti-cholesterol [10]. Currently, owing to the diverse biological activities of Ory, there is increasing interest in its application as a nutritional supplement. However, as a hydrophobic compound, low absorption and poor bioaccessibility severely limit its application. Interestingly, studies have shown that a fraction of Ory can be absorbed into the plasma in its intact form after ingestion [11]. Therefore, to exert beneficial health effects, Ory must be effectively transported and distributed to the tissues. Based on this evidence, we hypothesised that Ory could reversibly bind to the plasma protein HSA, inducing structural changes in HSA that subsequently influence the efficient delivery of Ory. Consequently, elucidating the molecular mechanisms of Ory binding to HSA, along with the release of Ory from HSA, has become a topic of considerable interest.

However, to the best of our knowledge, existing studies on the HSA-Ory system have primarily focused on characterising its binding interactions, such as binding sites, binding constants, and interaction forces [1]. In contrast, investigations of the binding process, binding stability, and dynamic behaviour of ligand dissociation remain limited. Therefore, in addition to characterising the interaction between HSA and Ory using multispectral analysis and molecular docking, this study integrated molecular dynamics and steered molecular dynamics simulations to elucidate the binding mechanism, binding stability, dissociation behaviour, and conformational changes of the complex. These findings provide a more comprehensive understanding of the molecular mechanisms underlying non-covalent interactions within the HSA-Ory complex and may offer useful insights for future studies on the applications of Ory.

## 2. Materials and Methods

### 2.1. Materials

HSA was purchased from Solarbio (Beijing, China). Ory and Digitoxin (Dig) were purchased from MedChemExpress (Monmouth Junction, NJ, USA). Warfarin sodium (War) and Ibuprofen (Ibu) were purchased from Aladdin (Shanghai, China). All the chemicals and solvents used in this study were of analytical grade. Tris-HCl buffer (20 mM) was prepared and used to dissolve HSA to obtain a stock solution (10 μM). Ory was initially dissolved in DMSO to prepare a stock solution (10 mM), which was then diluted with buffer to the desired concentration. The final concentration of DMSO in all the working solutions was maintained below 0.1% (*v*/*v*).

### 2.2. UV-Vis Spectroscopy

HSA was diluted to 2 μM, and its UV-Vis absorption spectra (both in the absence and presence of Ory) were recorded over the wavelength range of 200–400 nm using a UV-1200 spectrophotometer (Meipuda Instrument Co., Ltd., Shanghai, China).

### 2.3. Fluorescence Spectroscopy

HSA (2 μM) was combined with different concentrations of Ory. The mixtures were kept for 15 min at various temperatures. Their fluorescence spectra were recorded at 2.5 nm slit width and an excitation wavelength of 280 nm. Moreover, to eliminate the impact of filter effects within the system on data accuracy, the fluorescence intensity was corrected using the following formula:

(1) $$F_{cor}=F_{obs}\times 10^{(A_{ex}+A_{em})÷2}$$

where *F_cor_* and *F_obs_* represent the fluorescence intensities after and before correction, respectively, and *A_ex_* and *A_em_* denote the absorbance values at the excitation and emission wavelengths, respectively.

The Stern-Volmer equation was used to analyse the quenching mechanism [12]:

(2) $$\frac{F_{0}}{F}=1+K_{sv}\left[Q\right]=1+k_{q}\tau_{0}\left[Q\right]$$

In this context, *F* and *F*_0_ represent the fluorescence intensities of HSA in the presence and absence of Ory, respectively. The quenching constant is represented by *K_sv_*, with [*Q*] referring to Ory concentration, *k_q_* is the quenching rate constant, and *τ*_0_ is the average lifetime of the fluorophore (10^−8^ s) [12]. The binding constant (*K_a_*) and the number of binding sites (*n*) were determined using the following equation [13]:

(3) $$\log\frac{F_{0}-F}{F}=\log K_{a}+n\log\left[Q\right]$$

To gain insights into the thermodynamics of the HSA-Ory interaction, the Van’t Hoff Equation (3) and the associated thermodynamic relationship (4) were applied to derive key parameters such as Δ*H*, Δ*S*, and Δ*G*:

(4) $$\ln K_{a}=-\frac{\Delta H}{RT}+\frac{\Delta S}{R}$$

(5) ΔG=ΔH−TΔS

Here, *T* values of 298, 304, and 310 K were used with *R* = 8.314 J × mol^−1^ × k^−1^ [14].

In addition, the synchronous fluorescence spectra of the samples were recorded by setting the wavelength intervals between excitation and emission (Δλ = λ_em_ − λ_ex_) to 15 nm and 60 nm, respectively. Three-dimensional fluorescence spectra were acquired at excitation wavelengths spanning 200–350 nm and emission wavelengths recorded from 250–500 nm, respectively. The measurements were performed using a slit width of 2.5 nm and scanning speed of 2400 nm/min.

### 2.4. Investigation of Binding Sites

Different site probes, War, Ibu, and Dig, were used for sites I, II, and III, respectively [15]. HSA (2 μM) and Ory (8 μM) were initially prepared and incubated at room temperature for 10 min, followed by the addition of site markers at different concentrations and further incubation for 15 min. Fluorescence spectra were recorded at an excitation wavelength of 280 nm. The intrinsic fluorescence of the markers was subtracted and the inner-filter effects were corrected according to Equation (1). The probe displacement was calculated using the following equation [6]:

(6) $$Probe\;displacement\;\left(\%\right)=\frac{F_{2}}{F_{1}}\times 100$$

where *F*_2_ and *F*_1_ represent the fluorescence intensities of the HSA-Ory complex in the presence and absence of the specific probe, respectively. Subsequently, War (10 μM) was mixed with HSA (2 μM) and incubated for 10 min. Ory was then added at different concentrations, and the mixture was gently shaken and incubated for an additional 15 min. Fluorescence spectra were measured at an excitation wavelength of 280 nm.

### 2.5. Energy Transfer Between Ory and HSA

A 20 μM HSA solution and a 20 μM Ory solution were prepared. The UV-Vis spectra of Ory, as well as fluorescence spectra of both HSA and HSA-Ory mixtures, were measured with the same settings as described earlier. The energy transfer efficiency (*E*), donor-acceptor distance (*r*), Förster radius (*R*_0_), and spectral overlap integral (*J*) were determined using the following equations:

(7) $$E=\frac{{R_{0}}^{6}}{{R_{0}}^{6}+r^{6}}=\frac{F_{0}-F}{F_{0}}$$

(8) $${R_{0}}^{6}=8.8\times 10^{-25}K^{2}N^{-4}\varphi J$$

(9) $$J=\frac{\sum F\left(\lambda \right)\varepsilon \left(\lambda \right)\lambda^{4}\Delta \lambda }{\sum F\left(\lambda \right)\Delta \lambda }$$

Here, *K*^2^ (2/3) denotes the dipole spatial orientation factor, *N* (1.336) is the medium’s refractive index, *φ* (0.118) represents the donor’s fluorescence quantum yield, *F*(*λ*) is the fluorescence intensity of HSA at a given wavelength and *ε*(*λ*) is the molar absorbance of Ory [16].

### 2.6. FT-IR Spectroscopic Measurements

Mixtures of Ory and HSA, as well as individual HSA and Ory solutions, were freeze-dried. Subsequently, 2 mg of each sample was finely ground with KBr at a ratio of 1:100 (*w*/*w*) and compressed into pellets for analysis. The Fourier-transform infrared (FT-IR) spectra were recorded in the mid-infrared region (400–4000 cm^−1^) using a Tensor 27 FT-IR spectrometer (Bruker Optics, Karlsruhe, Germany). To ensure high spectral quality, each measurement was performed with a spectral resolution of 4 cm^−1^ and accumulated over 32 consecutive scans. The resulting spectra were baseline-corrected and analysed to identify the characteristic vibrational modes associated with protein secondary structure and ligand-induced conformational changes. Data analysis was performed using Peak Fit software (version 4.12).

### 2.7. Molecular Docking

The three-dimensional X-ray structure of HSA, corresponding to PDB entry 1HA2, was obtained from the RCSB Protein Data Bank (https://www.rcsb.org/, accessed on 3 Jauary 2025). Prior to computational analysis, all crystalline water molecules and bound ligands present in the original structure were stripped away. The chemical structure of Ory was retrieved from PubChem (CID: 5282164), and its three-dimensional structure was subsequently energy-minimised using the MMFF94 force field in Chem3D. In this study, site-competitive experiments were conducted to identify the primary binding site of Ory on HSA, which was determined to be site I. Molecular docking simulations were subsequently performed using AutoDock Vina (v1.2.7). The docking grid was centred at coordinates (32.551, 13.513, 9.638) Å, with a cubic box size of 30 Å along each axis (x, y, z) to fully encompass the target binding region. Default values were applied to all other settings. Prior to the main docking process, a re-docking procedure was conducted using warfarin, the original ligand, to confirm the accuracy and reliability of the docking methodology. Re-docking War into site I yielded an RMSD value of 0.129 Å (<2 Å) (Figure S1), validating the reliability of the used docking parameters. The pose with the most negative AutoDock Vina score from 100 independent runs was selected as the best docking conformation. The molecular docking results were visualised using DS 2024 Visualiser Client.

### 2.8. Molecular Dynamics (MD) Simulations

MD simulations of the HSA-Ory complex were performed using the best docking conformation from AutoDock Vina with GROMACS 2019.6 under the AMBER14SB force field. The ligand parameters were described by the General Amber Force Field (GAFF) using AM1-BCC charges. The systems were solvated in a cubic box filled with the TIP3P water model at a minimum distance of 1.0 nm from the solute to the box edge. Na^+^ and Cl^−^ ions were added to neutralize the system and achieve a salt concentration of 0.15 M. Electrostatic interactions were treated using the PME method with a cut-off of 1.0 nm, and the same cut-off was applied to the van der Waals interactions. The bonds involving hydrogen atoms were constrained using the LINCS algorithm. The temperature (300 K) and pressure (1 bar) were controlled using a V-rescale thermostat and a Parrinello–Rahman barostat, respectively. After energy minimisation (5000 steps), the system was equilibrated under the NVT and NPT ensembles (each 500 ps), followed by 100 ns production runs with a 2 fs time step. The binding free energy was calculated using the MM-PBSA method with 200 snapshots extracted every 10 ps from the last 20 ns, and convergence was verified based on energy stability.

### 2.9. Steered Molecular Dynamics (SMD) Simulations

After conventional MD simulations, SMD simulations were carried out according to a previous study [17] with some modifications to reveal the dissociation behaviour of Ory from HSA. The HSA-Ory complex conformation selected after the end of the 100 ns conventional MD simulation was placed in a water box with dimensions of (11.12, 11.12, 22.07) nm along the (x, y, z) axes, respectively. The pulling simulation was conducted over 400 ps using a harmonic spring with a force constant of 1000 kJ·mol^−1^·nm^−2^ and a constant pulling velocity of 0.01 nm·ps^−1^. The centre of mass of HSA was restrained, whereas Ory was pulled along the z-axis. The simulation generated 400 configurations along reaction coordinates. From them, 30 umbrella sampling (US) windows were selected at approximately equal intervals to ensure sufficient overlap between adjacent windows. After removing the external pulling force, each US window was subjected to a 10 ns equilibrium simulation under the above-described temperature and pressure control conditions. The first 2 ns of each trajectory were discarded as equilibration, and the remaining data were used for the analysis. Statistical errors were estimated using a Bayesian bootstrap analysis. A weighted histogram analysis method (WHAM) was employed to calculate the potential of the mean force (PMF) profile. The convergence of the PMF was assessed by comparing profiles obtained from different time intervals and by ensuring adequate overlap of histograms between neighbouring windows.

### 2.10. Statistical Analysis

All experiments were performed at least thrice. Data were expressed as mean ± standard deviation and were analysed using SPSS software (version 26). One-way analysis of variance (ANOVA) followed by Duncan’s multiple range test was performed to compare the means among groups, and *p* < 0.05 was considered statistically significant.

## 3. Results and Discussion

### 3.1. UV-Vis Spectroscopy

UV-Vis spectroscopy is a convenient and valuable analytical tool for studying ligand-protein interactions and monitoring protein structural changes [18]. The effects of various concentrations of Ory on HSA are reflected in the spectra shown in Figure 1B. HSA exhibits a characteristic absorption peak at 280 nm, resulting from π–π* transitions in its aromatic residues, namely, Tyr and Trp. As shown in Figure 1B, as the Ory concentration increased, HSA’s absorbance rose gradually, along with a red-shift. This indicated that the interaction between HSA and Ory induces conformational alterations in the protein and reduces the local polarity around aromatic amino acids. Furthermore, they indicated the potential formation of a complex between Ory and HSA [19].

### 3.2. Analysis of Fluorescence Quenching

**Figure 2:**
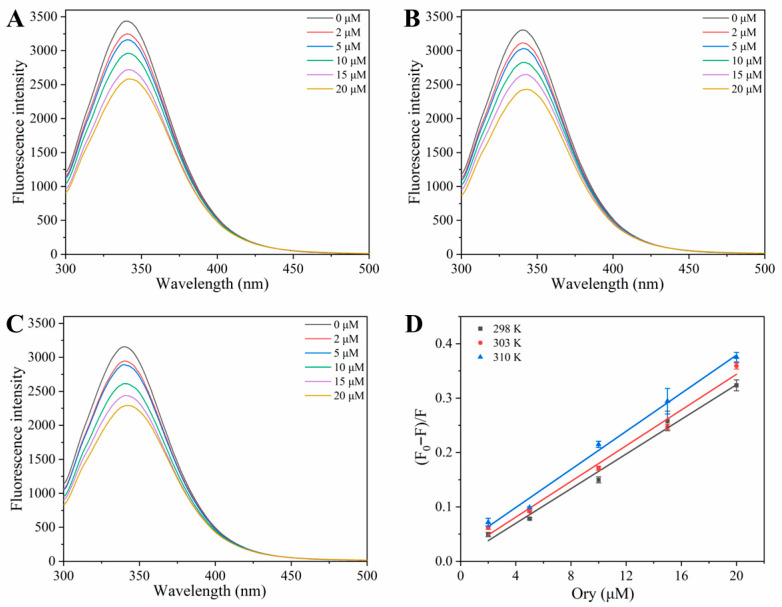
Fluorescence spectra of the interaction between HSA and Ory ((**A**) 298 K; (**B**) 304 K; (**C**) 310 K) and their Stern Volmer curves (**D**).

Fluorescence spectroscopy is a robust and sensitive approach that is commonly employed to investigate the interactions between proteins and low-molecular-weight compounds. This technique leverages the natural fluorescence of proteins, which mainly originates from aromatic side chains. Among them, the Trp and Tyr residues exhibited stronger fluorescence intensity than the Phe residues [16]. Figure 2A–C presents the fluorescence emission spectra of HSA with varying Ory concentrations under three different thermal treatments. It can be observed that increasing Ory quenched HSA fluorescence intensity in a concentration-dependent manner and caused the red-shift (from 340.6 to 345.4 nm at 298 K). This suggests that the interaction of Ory with HSA quenches the endogenous fluorescence of HSA, leading to conformational alterations of HSA and modifications in the local environment of Trp and Tyr residues [5].

Static quenching occurs when a non-fluorescent complex forms between the quencher and the protein in the ground state, thereby diminishing the observed fluorescence intensity. As such complexes are often thermally labile, an increase in temperature typically destabilises the association, resulting in a lower quenching constant [20]. Dynamic quenching occurs when the quencher collides with the excited protein molecules. As the temperature increases, intermolecular diffusion and collision become more intense, leading to an increase in the quenching constant. In combined quenching, both processes occur simultaneously. Figure 2A–C illustrate that the quenching rate of HSA changed in response to the addition of the same concentration of Ory at varying temperatures. When the concentration of Ory was held constant, the fluorescence intensity decreased with temperature, suggesting that a higher ambient temperature negatively affected the binding of HSA to Ory [21]. As shown in Figure 2D and Table 1, *K_sv_* increased with increasing temperature, a trend typically associated with dynamic (collisional) quenching. However, the calculated *k_q_* was substantially higher than the theoretical upper limit for diffusion-controlled collisions, suggesting a static quenching mechanism. These results suggest that the quenching mechanism may involve a combination of different pathways, indicating a mixed quenching process [22].

### 3.3. Binding Constants and Number of Binding Sites

**Figure 3:**
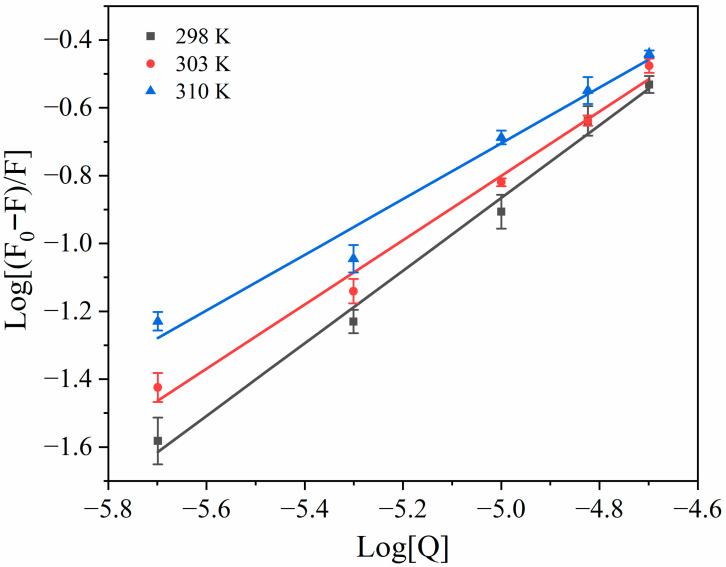
Double logarithmic plots of the interaction between HSA and Ory.

Table 2 presents the *K_a_* and *n* values calculated from the slopes and intercepts of the double logarithmic plots in Figure 3. The value of *n* was approximately 1 at all tested temperatures, suggesting that Ory primarily interacts with HSA at one site. Site competition and molecular docking experiments demonstrated that Ory binds to the HSA site I. Additionally, the *K_a_* values ranged between 10^3^ and 10^5^ (L mol^−1^), suggesting a moderate binding affinity between HSA and Ory. Dai et al. [23] also reported a moderate binding between BSA and Ory, which is consistent with our findings. Furthermore, as the temperature increased, the *K_a_* values decreased, indicating that the binding between HSA and Ory became less stable at higher temperatures. This also suggests that the binding is an exothermic reaction, which is consistent with thermodynamic analysis [24].

### 3.4. Analysis of Thermodynamic Characteristics and Binding Forces

It has been demonstrated that small molecules typically engage with proteins through a range of non-covalent interactions, including hydrogen bonds, electrostatic attractions, and others [14]. Thermodynamic parameters can be employed to ascertain the binding process and the principal force between proteins and small molecules. Δ*G* < 0 suggests that this binding is spontaneous. When both Δ*H* and Δ*S* are negative, the interaction is mainly governed by hydrogen bonding or van der Waals forces. In contrast, if Δ*H* and Δ*S* are both positive, hydrophobic interaction becomes the primary force behind the interaction [25]. As illustrated in Table 3, Δ*G* between HSA and Ory was less than zero, and the Δ*H* and Δ*S* were also less than zero. These findings revealed that the association between HSA and Ory is likely spontaneous and exothermic, with van der Waals forces and hydrogen bonding potentially being the dominant intermolecular interactions. In addition, as a protein with multiple binding sites, HSA may interact with ligands through the cooperative contribution of several weak interactions, which could lead to the amplification of thermodynamic parameter values.

### 3.5. Synchronous Fluorescence Spectroscopy

**Figure 4:**
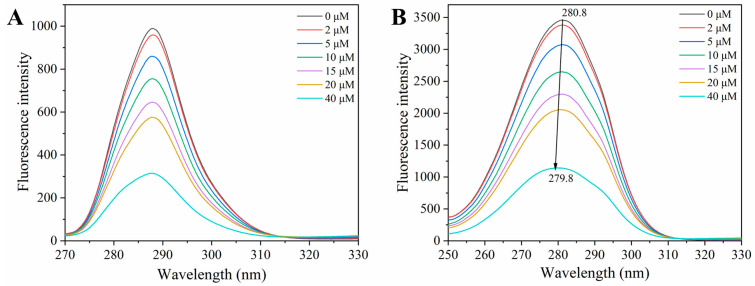
Synchronous fluorescence spectrum ((**A**) represents Δλ = 15 nm, (**B**) represents Δλ = 60 nm).

Synchronous fluorescence spectroscopy is useful for evaluating microenvironmental alterations surrounding protein fluorophores. Specifically, setting the wavelength offset (Δλ) to 15 nm preferentially targets Tyr residues, while a Δλ of 60 nm targets Trp [26]. In comparison, it can be clearly observed from Figure 4 that the emission intensity at Δλ = 60 nm (3458) was substantially greater than that at Δλ = 15 nm (989), corroborating previous reports that Trp dominates the intrinsic fluorescence of HSA [1]. As demonstrated in Figure 4A,B, increasing concentrations of Ory led to the progressive quenching of fluorescence in both Tyr and Trp. Under Δλ = 60 nm conditions, a blue shift (from 280.8 to 279.8 nm) could be observed, implying that the microenvironment around Trp residues was altered owing to their interactions with Ory [3]. HSA possesses a single tryptophan residue, Trp214, located in site I. Critically, both molecular docking and MD simulations consistently revealed π-π stacking interactions between Trp214 and Ory, corroborating the experimental findings.

### 3.6. Three-Dimensional Fluorescence Spectroscopy

**Figure 5:**
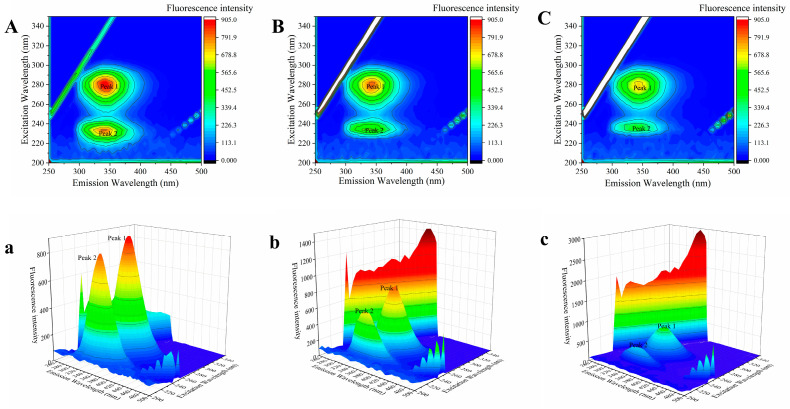
Three-dimensional fluorescence spectra of HSA-Ory ((**A**,**a**): C_HSA_ = 2 μM, (**B**,**b**): C_HSA_ = 2 μM, C_Ory_ = 20 μM, (**C**,**c**): C_HSA_ = 2 μM, C_Ory_ = 40 μM).

Three-dimensional fluorescence spectroscopy enables simultaneous identification and characterisation of multicomponent complexes by monitoring fluorescence intensity variations during synchronous scanning of excitation and emission wavelengths [27]. This technique provides a robust approach to detect conformational changes in proteins with enhanced sensitivity. The three-dimensional fluorescence spectrogram in Figure 5 shows two main characteristic peaks (Peak 1 and Peak 2). Peak 2 (230 nm/340 nm) corresponds to the characteristic C=O emission of the polypeptide backbone, which is related to the secondary structure of HSA. The strong fluorescence peak 1 (275 nm/340 nm) primarily represents the fluorescence signature of aromatic residues, particularly Trp and Tyr [15]. Table S1 completely displays the corresponding characteristic peak values. Upon the addition of Ory, a significant decline in the fluorescence intensity of the two characteristic peaks was detected (Figure 5 and Table S1). The fluorescence intensity of Peak2 changed from 808.8 to 416.9, while that of Peak1 decreased from 894.3 to 703.4 (Table S1). The change in Peak 1 suggests that Ory binding perturbs the local microenvironment surrounding Trp and Tyr residues. Meanwhile, Peak 2 exhibited a blue shift, indicating that Ory might induce conformational changes in HSA, particularly affecting its secondary structure [18].

### 3.7. Energy Transfer Between Ory and HSA

**Figure 6:**
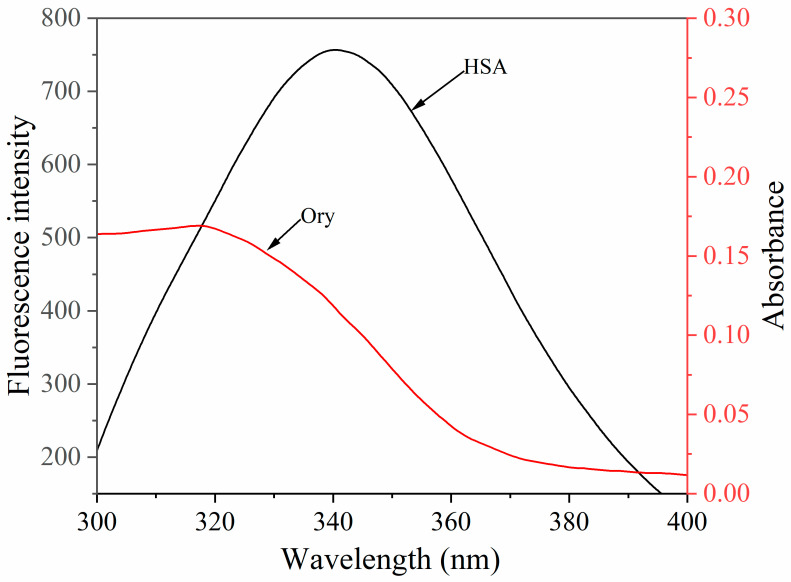
The overlap between the fluorescence spectrum of HSA and the UV spectrum of Ory.

Förster resonance energy transfer (FRET) is a useful technique for elucidating complex formation by measuring binding distances [8]. According to the Förster theory, energy transfer requires two key conditions: a significant overlap between the donor’s emission spectrum and the acceptor’s absorption profile and a donor-acceptor separation of less than approximately 7 nm. As shown in Figure 6, a sufficient spectral overlap was observed. Calculated from Equations (7)–(9), *J* = 1.21 × 10^−14^ cm^3^ L mol^−1^, *E* = 24.6%, *R*_0_ = 2.53 nm, *r* = 3.05 nm. The *r* value is clearly below 7 nm and comparable to *R*_0_. Collectively, these parameters indicate effective non-radiative energy transfer from HSA (donor) to Ory (acceptor), suggesting that the two molecules are in close proximity and may form a complex [8].

### 3.8. The Binding Location of Ory and Its Binding Mechanism with HSA

**Figure 7:**
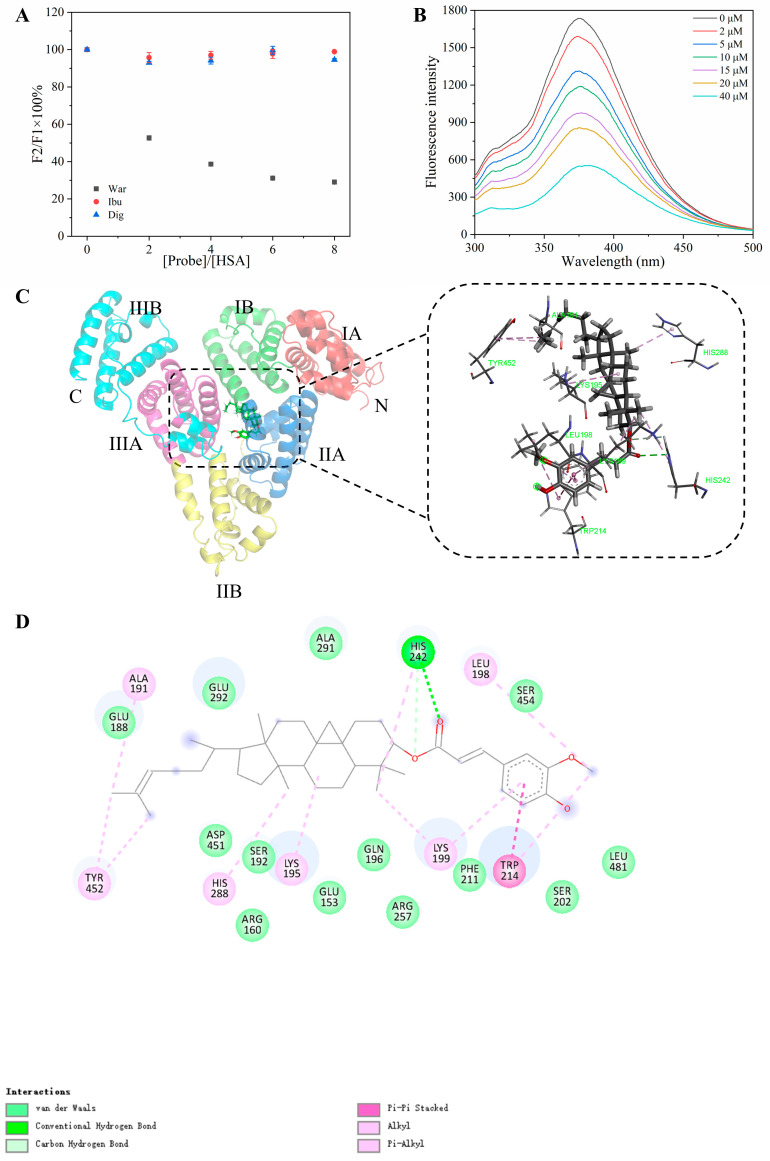
Impact of site-specific probes on the fluorescence of the HSA-Ory complex (**A**), fluorescence spectra of the HSA-War complex with increasing Ory concentrations (**B**), and the optimal docking conformation between HSA and Ory along with the 3D interaction diagram (**C**) and the 2D interaction diagram (**D**).

To identify the binding location of Ory on HSA, we conducted competitive substitution experiments using the site probes War, Ibu, and Dig at target sites I, II, and III, respectively. As illustrated in Figure 7A, the addition of War resulted in a progressive decrease in fluorescence intensity, whereas Ibu and Dig induced negligible changes. This indicates that Ory mainly competes with War for binding site I on HSA [28]. Subsequently, to further confirm the binding site of Ory on HSA, it was introduced into the War-HSA system [15]. As shown in Figure 7B, the addition of Ory caused a reduction in fluorescence intensity, suggesting the competitive displacement of War from its binding site on HSA. This supports the conclusion that Ory binds to site I of HSA and is consistent with the previous finding that Ory has only one binding site on HSA.

Molecular docking is a widely used computational approach to predict the most likely binding conformations between proteins and small molecules [29]. With a docking score of −10.028, the best conformation of the HSA-Ory complex and their interaction patterns are presented in Figure 7C,D. Consistent with competitive experimental results, the docking results indicated that Ory was effectively accommodated within site I of HSA. Within the binding site, Ory interacted with Trp214 through a π–π stacking interaction involving its aromatic ring (Figure 7D), a contact that likely underlies the observed quenching of HSA’s intrinsic fluorescence upon Ory binding. Some studies have suggested that the addition of aromatic rings contributes to an increase in the affinity [30]. In addition, Ory forms a stable complex within the binding pocket, primarily stabilised by a crucial hydrogen bond between His242 and the ligand, which is pivotal for anchoring the molecule to the active site. Ory also forms hydrophobic interactions with surrounding residues such as His288, which collectively contribute to the stability of the complex. Subsequent computational simulations revealed that residues such as Lys199, Lys195, and His288 played critical roles in regulating the dissociation behaviour of Ory.

### 3.9. FT-IR Spectroscopic Analysis

FT-IR spectroscopy is widely used to characterise protein functional groups and secondary structures. HSA has three distinct amide bands: Amide A near 3226 cm^−1^, Amide I in the range of 1600–1700 cm^−1^, and Amide II between 1500–1600 cm^−1^. Among them, the Amide I band is the most sensitive to alterations in protein secondary structure. The changes in the amide bands were mainly due to O–H/N–H stretching, C=O stretching, and N–H bending coupled with C–N stretching [31].

**Figure 8:**
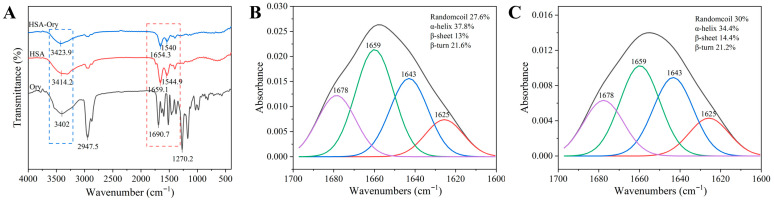
FT-IR spectra of HSA, Ory and HSA-Ory (C_HSA_ = 2 μM, C_Ory_ = 40 μM) (**A**), The curve-fitted amide I region of HSA (**B**) and HSA-Ory (**C**).

After adding Ory to HSA (Figure 8A), three amide bands exhibited significant shifts, such as the Amide I band shifted from 1659.1 to 1654.3 cm^−1^. Previous studies have shown that ligand binding to proteins via hydrophobic interactions can affect peaks in the Amide I region [32]. Therefore, the changes in the absorption peak intensity within this region upon Ory addition suggest the possible involvement of hydrophobic interactions. Furthermore, the shift observed in Amide A may indicate the formation of hydrogen bonds between Ory and the O–H groups in HSA. To further investigate the effect of Ory addition on the secondary structure of HSA, the Amide I region was analysed (Figure 8B,C). The results indicate that upon the addition of Ory, the *α*-helix and *β*-turn content of HSA decreased, while the proportion of *β*-sheets and random coils increased. These findings suggest that Ory binding induces secondary structural changes in HSA.

### 3.10. Binding Stability

**Figure 9:**
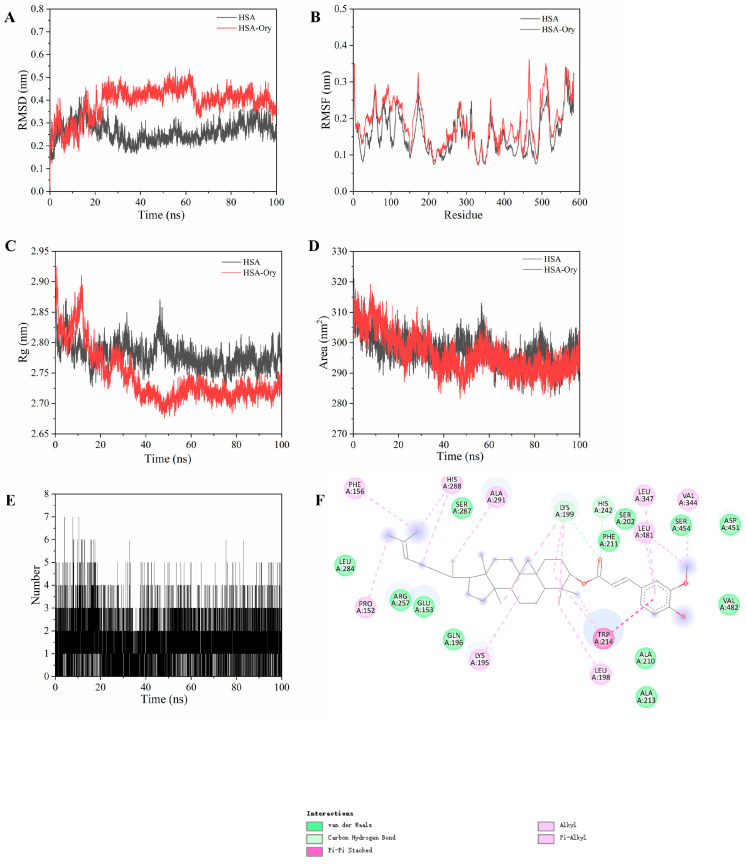
RMSD (**A**), RMSF (**B**), Rg (**C**), and SASA (**D**) values of HSA with and without Ory. The hydrogen bond number between HSA and Ory throughout the whole MD simulation (**E**). Two-dimensional interaction diagram between HSA and Ory of the last frame extracted from MD simulation (**F**).

To further investigate the binding behaviour of HSA to Ory, MD simulations were conducted for both unliganded HSA and the HSA-Ory complex. To assess the conformational stability and dynamic behaviour of HSA, the root mean square deviation (RMSD) was calculated in the presence and absence of Ory [25]. According to many studies, a simulation system is generally considered to have reached dynamic equilibrium when RMSD fluctuation is less than 2.00 Å [33]. Figure 9A shows that after approximately 23 ns, both free HSA and the HSA-Ory complex reached dynamic equilibrium, confirming that Ory could form a stable complex with HSA in a dynamic environment [34]. Free HSA maintained lower RMSD fluctuations of 0.20–0.35 nm, while the HSA-Ory complex exhibited higher fluctuations of 0.35–0.50 nm. These results suggest that Ory binding induces local conformational flexibility without compromising the global structural integrity of HSA. Root mean square fluctuation (RMSF) is a key parameter for assessing the flexibility of individual amino acid residues within a protein. As shown in Figure 9B, the reduced fluctuations of residues in subdomain IIA indicate that upon binding with Ory, the rigidity at site I of HSA increased relative to other regions [34]. Additionally, the radius of gyration (Rg) was calculated to evaluate the effect of Ory binding on HSA compactness. Binding of Ory decreased the Rg of HSA, indicating a more compact conformation (Figure 9C). The HSA-Ory complex exhibited a slightly reduced average Rg of 2.72 nm compared to 2.78 nm for unbound HSA. These values are consistent with those reported in previous studies [5], indicating that the HSA structure becomes more stable and tightly folded upon Ory binding. Solvent-accessible surface area (SASA) quantifies solvent exposure and serves as a probe for protein structural dynamics. As depicted in Figure 9D, the HSA-Ory complex exhibited a reduced SASA compared to free HSA, particularly after ~30 ns. The average SASA values for free HSA and the complex were 296 and 293 nm^2^, respectively. This suggests that the folding of HSA is altered upon binding of Ory, disturbs the local environment of amino acid residues, decreases the solvation capacity of the protein, and supports the idea that Ory causes protein densification [35].

**Figure 10:**
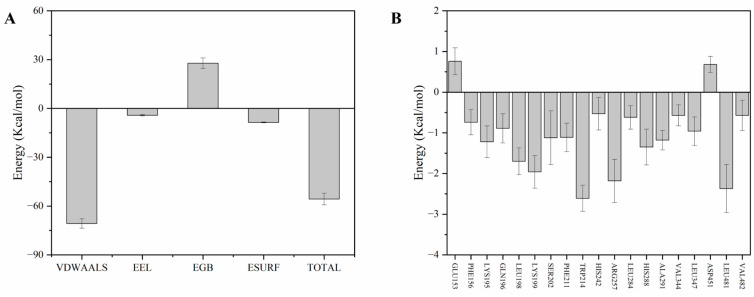
Binding free energy and its individual components (**A**). The energy contribution of key residues (**B**).

Intermolecular hydrogen bonds between proteins and ligands are critical for stabilising complexes [36]. As illustrated in Figure 9E, the HSA-Ory complex consistently maintained at least one hydrogen bond throughout the simulation, reflecting its enhanced stability. A similar binding behaviour was observed for myricitrin interacting with HSA [37]. The last frame was extracted to analyse the protein-ligand binding pattern. As displayed in Figure 9F, it can be assumed that a new hydrogen bond was formed during the MD simulation between Ory and the key residues of HSA, such as Lys199. Moreover, the MM-PBSA decomposition results presented in Figure 10B highlighted Lys199 as a key contributor to the binding stability of the HSA-Ory complex. Overall, these findings indicate that intermolecular hydrogen bonding is essential for stable ligand binding in dynamic simulation environments.

### 3.11. MM-PBSA Analysis

MM-PBSA analysis yielded a binding free energy of −56.72 kcal/mol for the HSA-Ory complex (Figure 10A), suggesting that Ory binds spontaneously to HSA, resulting in a stable interaction. Additionally, the binding free energy was further analysed by dividing it into four components to better understand the interaction energies that make key energetic contributions to the binding process. Among the favourable energy components, van der Waals interactions made the largest contribution, highlighting their dominant role in stabilising Ory within the HSA binding pocket and serving as the primary driving force for the formation and maintenance of the HSA-Ory complex. Similar binding behaviour has been reported for various small molecules interacting with HSA; for instance, Tong et al. found that the main force between ferulic acid ethyl ester and HSA is the van der Waals interaction [25]. Furthermore, the total binding free energy was decomposed into individual HSA residues to determine which residues had a significant impact on maintaining binding stability [18]. The results are shown in Figure 10B. Residues such as Trp214, Arg257, and Leu481 (the absolute value was higher than 2 Kcal/mol) contributed significantly to the binding process. Combining the results of molecular docking and MD simulation, Ory interacts with these residues through π–π stacking and van der Waals forces and also forms alkyl interactions with Leu481 during the simulation process. Together, these interactions enhance the overall binding stability of the complex.

### 3.12. PCA and FEL Analysis

**Figure 11:**
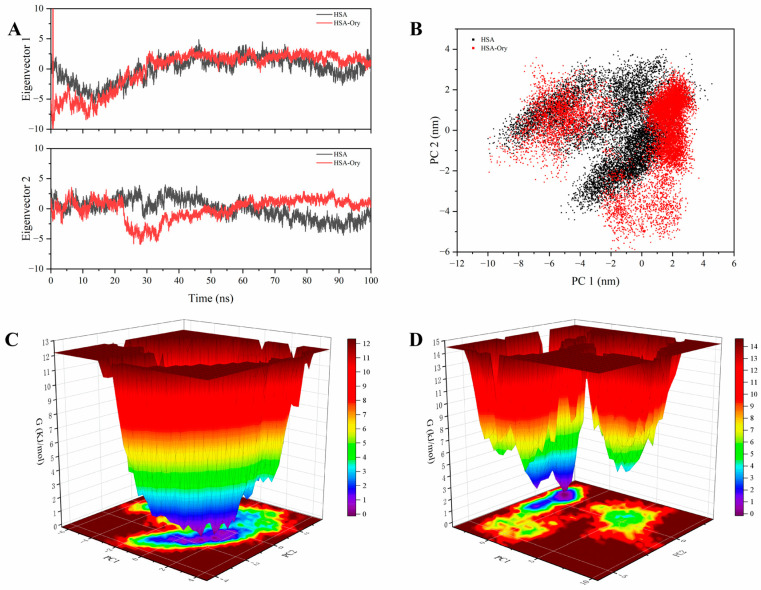
Two-dimensional projections of PC1 and PC2 of free state and Ory-bound HSA (**A**). Conformational projections on eigenvector 1 and eigenvector 2 over time (**B**). The Gibbs energy landscapes for native HSA (**C**) and HSA-Ory complex (**D**).

Principal component analysis (PCA), which reduces data dimensionality through the computation of eigenvectors and their corresponding eigenvalues, is helpful for understanding the influence of ligand interactions on the collective motion of proteins during MD simulations [38]. The two-dimensional projection of the first and second principal components (PC1 and PC2) derived from HSA is illustrated in Figure 11A. The results revealed that unliganded HSA occupies a wider phase space than HSA-Ory, indicating a broader conformational ensemble and more diverse structural transitions of HSA without a ligand. On the contrary, the more compact phase space of the HSA-Ory complex implied that the collective motions of the complex are limited to a specific and tighter subspace [38,39]. Figure 11B shows the projections of the first two eigenvectors over time, implying a unique structural arrangement for HSA-Ory, in contrast to native HSA [39]. Free energy landscape (FEL) analysis derived from PCA provided valuable insights into the conformational transitions of proteins associated with ligand binding [40]. As shown in Figure 11C,D, the deeper blue zones in the FEL plots correspond to more stable conformational states with lower energy levels. The HSA-Ory complex occupied a single global energy minimum located within a broad energy basin, in contrast to the apo form of HSA, which displayed two distinct energy wells. This difference likely results from non-covalent interactions between HSA and Ory driven by electrostatic forces, hydrogen bonding, and hydrophobic effects, which stabilise the protein in a more constrained and favourable conformation [38]. Figure S2 shows the minimum energy conformations of the HSA-Ory complex from FEL, revealing tight accommodation of Ory within the HSA binding site, with persistent interactions involving Lys199, His242, Trp214, and Leu481. Collectively, the PCA and FEL results demonstrated that HSA complexed with Ory is more stable than apo-HSA and adopts a thermodynamically stable conformational state.

### 3.13. Unbinding Behaviour and PMF Calculation

**Figure 12:**
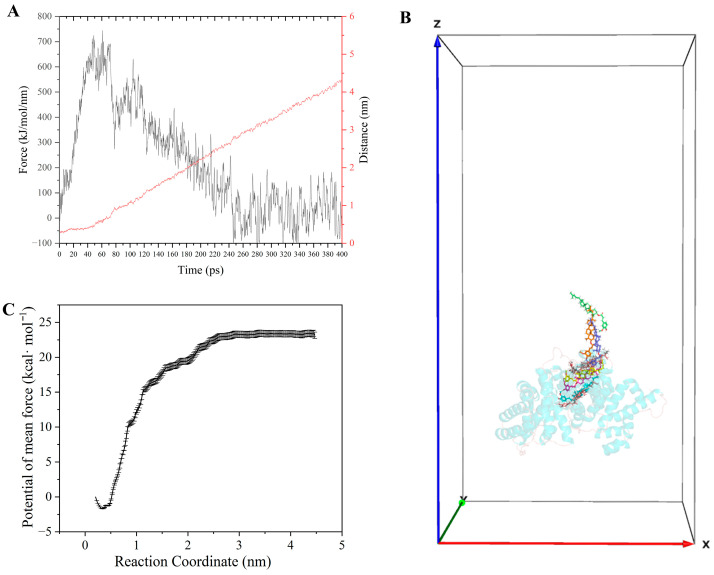
Pulling force (black, left y axis) and distance between HSA and Ory over time (red, right y axis) (**A**). Dissociation pathway for Ory from HSA protein during SMD simulation (**B**). PMF curve obtained from the US based on WHAM analysis (**C**).

To further investigate the dissociation behaviour of Ory from HSA, SMD simulations and US algorithms were performed to analyse the detailed unbinding process and calculate the unbinding free energy. As illustrated in Figure 12A, the force exerted on the spring progressively increases, reaching approximately 745 kJ mol^−1^ nm^−1^ at around 61 ps. Figure S3 illustrates the interactions between Ory and the surrounding key amino acid residues within the binding pocket during dissociation. The accumulated force ultimately overcame the non-covalent interactions that stabilised the Ory ligand at its binding site on HSA, such as hydrogen bonding with Lys199 and His242 (Figure S3A). Simultaneously, an increase in the distance gradient was observed, indicating that Ory began moving away from its initial binding site on the protein. During the dissociation process, new hydrogen bonds were formed between Ory and residues of HSA, such as Ser202 (Figure S3B), Asp451, and Trp214 (Figure S3C). In addition to Lys195 and Lys199, the entrance to Sudlow’s site I cavity is surrounded by two other basic amino acid residues, Arg218 and Arg222 [41]. Around 128 ps, detectable non-covalent interactions, including hydrogen bond and π–cation interaction, formed between Ory and residues Arg218/Arg222 (Figure S3D–G). These interactions ruptured approximately 64 ps later, at ~192 ps. His288 was the last residue that maintained contact with the Ory ligand via hydrogen bonding (Figure S3H,I), and no further ligand-protein interactions were observed. In summary, certain residues, including the four located at the pocket entrance, play important roles in regulating the release of Ory from HSA proteins. Further, US was employed to estimate the free energy differences (ΔG) across each sampling window, enabling the reconstruction of the PMF along the defined reaction coordinate. The PMF profile was calculated using weighted histogram analysis. As shown in Figure S4, sufficient overlap among the sampling windows along the reaction coordinate ensured a reliable PMF curve for the ligand dissociation process [42]. The PMF curve (Figure 12B,C) indicated an energy barrier of 24.15 kcal/mol for the dissociation of the Ory molecule from Sudlow’s site I of HSA. This energy barrier is higher than that reported for HSA binding to nitric oxide drugs [17]. According to previous studies, HSA can be regarded as an important carrier protein for a variety of bioactive compounds [43]. The molecular-level insights obtained in this study contribute to a better understanding of the dissociation behaviour of Ory from HSA.

## 4. Conclusions

In conclusion, the integration of multi-spectroscopic experimental and computational approaches revealed that Ory can stably bind to site I of HSA through non-covalent interactions with several important surrounding residues. Results from three-dimensional fluorescence, synchronous fluorescence, and FEL analyses further suggested that Ory binding may induce conformational changes in HSA and alter the microenvironment of the residues interacting with Ory. Furthermore, conventional MD and SMD simulations provided atomic-level insights into the dynamic binding and dissociation behaviour between Ory and HSA, revealing the intermolecular non-covalent forces and key amino acid residues that govern their interaction. Overall, our study provides comprehensive insights into the molecular mechanisms underlying the non-covalent interactions between HSA and Ory.

## Figures and Tables

**Table 1:** Quenching rate constants and quenching constants of the interaction between HSA and Ory at different temperatures.

*T* (K)	*k_q_* (×10^12^ L mol^−1^ s^−1^)	*K_sv_* (×10^4^ L mol^−1^)	R^2^
298	1.63 ± 0.04 ^a^	1.63 ± 0.04 ^a^	0.9968
304	1.75 ± 0.01 ^b^	1.75 ± 0.01 ^b^	0.9954
310	1.95 ± 0.03 ^c^	1.95 ± 0.03 ^c^	0.9941

Annotation: Different letters indicate significant differences within the same group (*p* < 0.05).

**Table 2:** The binding constants and binding sites of HSA and Ory.

*T* (K)	*K_a_* (×10^4^ L mol^−1^)	*n*	R^2^
298	3.22 ± 0.94 ^a^	1.07	0.9919
304	0.90 ± 0.28 ^b^	0.95	0.9891
310	0.27 ± 0.09 ^b^	0.82	0.9737

Annotation: Different letters indicate significant differences within the same group (*p* < 0.05).

**Table 3:** Thermodynamic parameters at different temperatures.

*T* (K)	Δ*H* (kJ mol^−1^)	Δ*S* (J mol^−1^ k^−1^)	Δ*G* (kJ mol^−1^)
298	−160.28	−451.8	−25.63 ± 0.81 ^a^
304			−22.95 ± 0.75 ^b^
310			−20.21 ± 0.93 ^c^

Annotation: Different letters indicate significant differences within the same group (*p* < 0.05).

## Data Availability

The original contributions presented in the study are included in the article/Supplementary Materials, further inquiries can be directed to the corresponding authors.

## Supplementary Materials

The following supporting information can be downloaded at: https://www.mdpi.com/article/10.3390/foods15081420/s1, Table S1: Characteristics of three-dimensional fluorescence spectra for the interaction between HSA and Ory. Figure S1: The docking conformation between HSA and War. Figure S2: 2D interaction diagram of the lowest energy conformations of the HSA-Ory complex from the FEL. Figure S3: Interaction diagram of Ory with surrounding key amino acids in the binding pocket during the dissociation process. Figure S4: Overlap of sampling windows along the reaction coordinate.
