# Supplementary material for: Unravelling the Interaction Mechanism Between Oryzanol and Human Serum Albumin: An Integrated Approach Using Multispectral Analysis and Molecular Simulations
Source: Foods. 2026 Apr 18;15(8):1420. doi: 10.3390/foods15081420 (PMC13114880; doi:10.3390/foods15081420)
Supplement: Supplementary file 1 [file foods-15-01420-s001.zip › foods-4227434-supplementary.pdf]

Table S1. Characteristics of three-dimensional fluorescence spectra for the interaction between HSA and Ory.

Systems	Peaks	$\lambda_{ex}/\lambda_{em}$ (nm)	$\Delta\lambda$ (nm)	F (a.u.)
HSA	Peak 1	275/340	65	894.3
	Peak 2	230/340	110	808.8
HSA + Ory 20 μM	Peak 1	280/340	60	811.5
	Peak 2	230/340	110	593.7
HSA + Ory 40 μM	Peak 1	275/340	65	703.4
	Peak 2	230/335	105	416.9

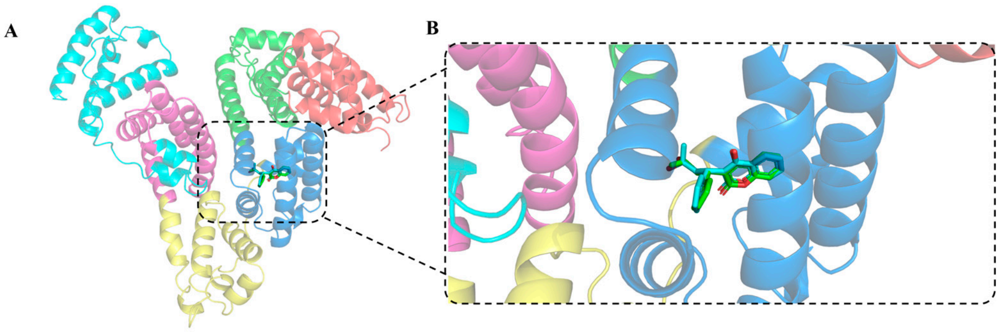

Figure S1. The docking conformation between HSA and War.

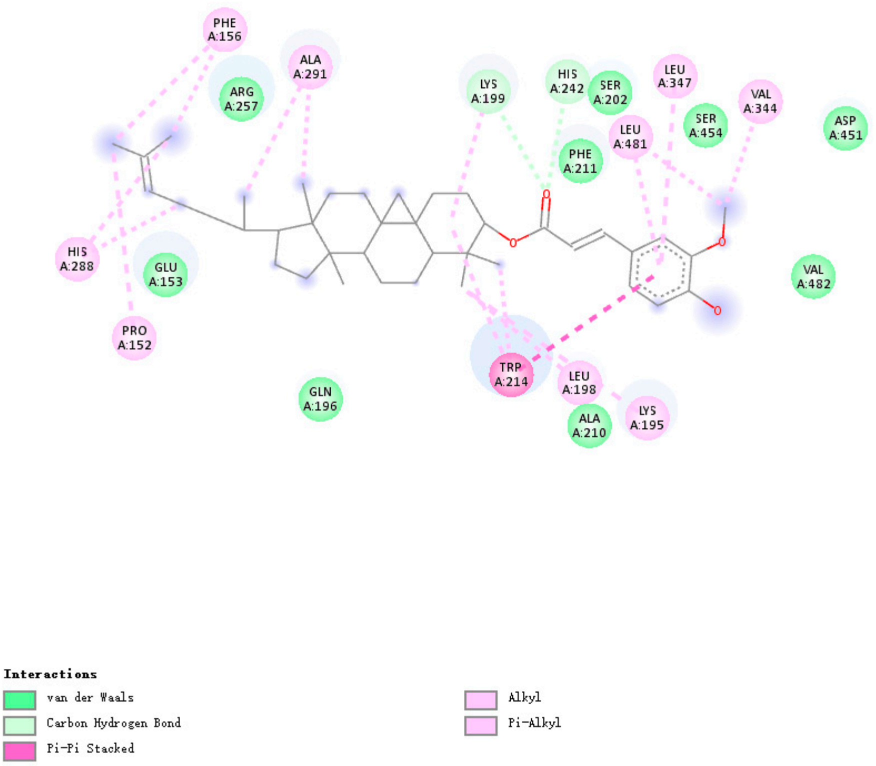

Figure S2. 2D interaction diagram of the lowest energy conformations of the HSA-Ory complex from the FEL.

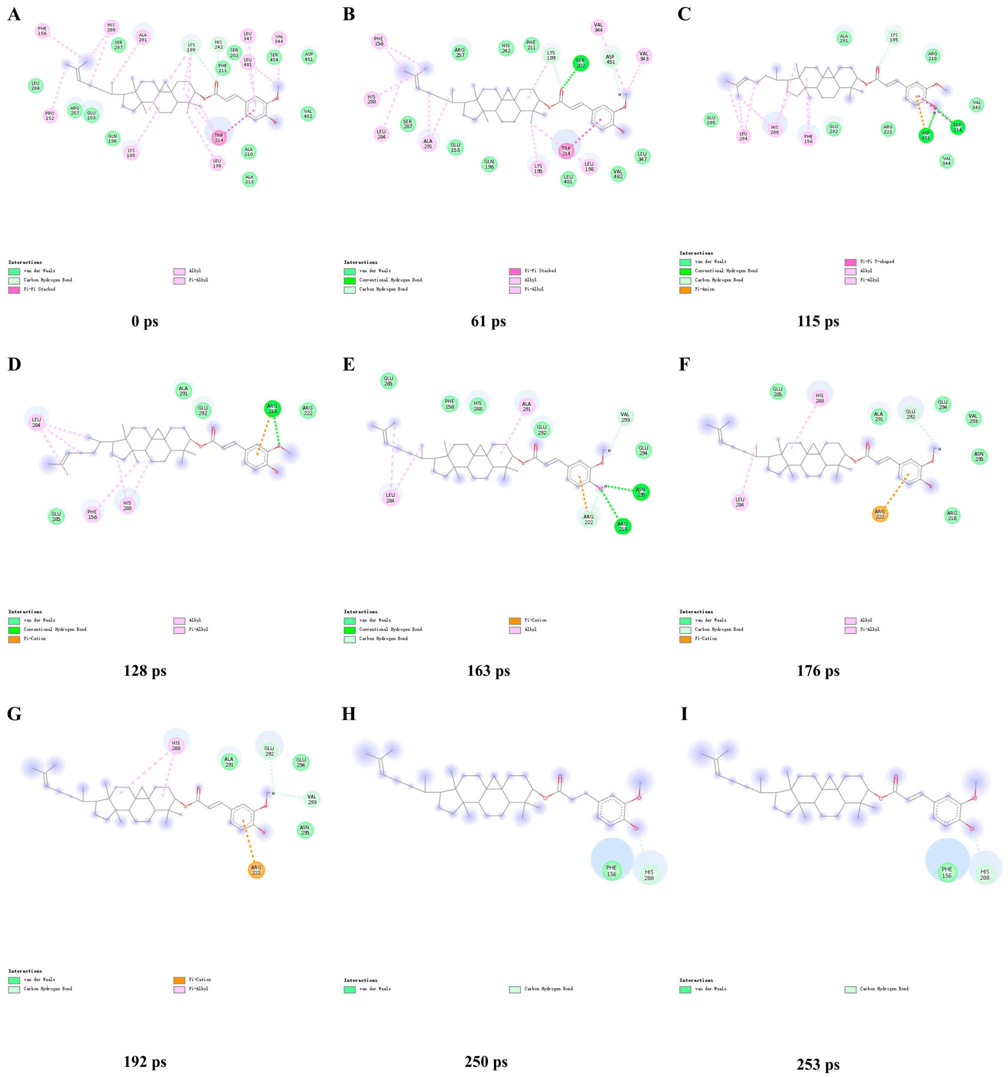

Figure S3. Interaction diagram of Ory with surrounding key amino acids in the binding pocket during the dissociation process.

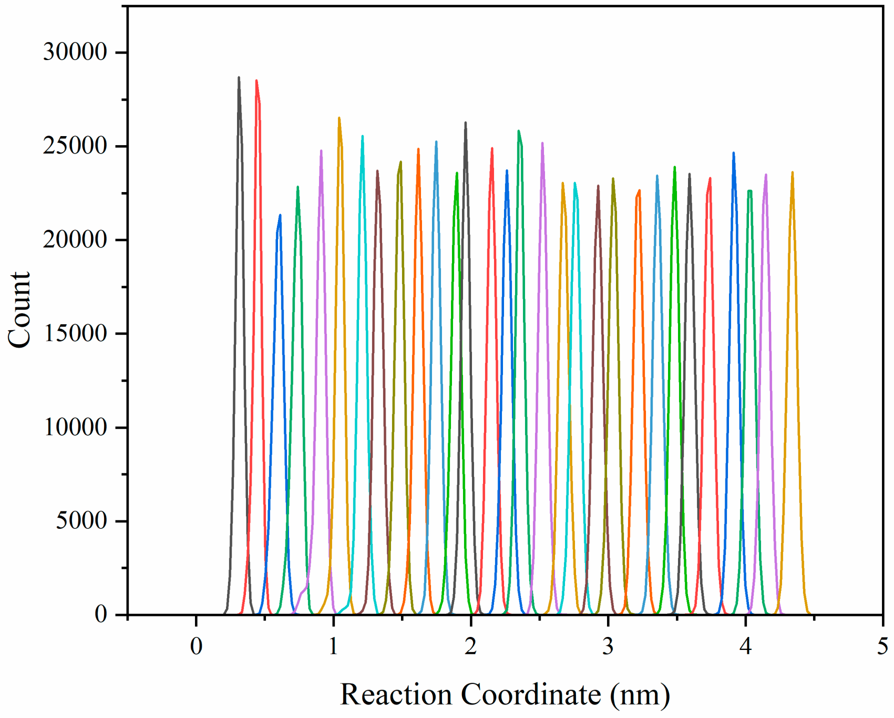

Figure S4. Overlap of sampling windows along the reaction coordinate.
